# HERV-K Envelope Induce a Humoral Response in Non-Hodgkin Lymphoma Patients

**DOI:** 10.1007/s00284-025-04511-8

**Published:** 2025-10-24

**Authors:** Ilaria Cossu, Stefano Ruberto, Elisabetta Filippi, Elena R. Simula, Marta Noli, Anna Mottula, Claudio Fozza, Leonardo A. Sechi

**Affiliations:** 1 Department of Biomedical Sciences, Section of Microbiology and Virology, University of Sassari, Viale San Pietro 43B, 07100 Sassari, Italy; 2 Environmental Factors in Degenerative Diseases Research Group, Instituto de Investigaciòn Sanitaria del Hospital Clìnico San Carlo (IdISSC), Madrid, Spain; 3 Department of Medicine, Surgery and Pharmacy, University of Sassari, Sassari, Italy; 4 Struttura Complessa Microbiologia e Virologia, Azienda Ospedaliera Universitaria Sassari, 07100 Sassari, Italy

## Abstract

Haematological malignancies represent a heterogenous group of diseases, encompassing lymphomas, leukaemia, and multiple myeloma. Among these, the involvement of human endogenous retroviruses (HERVs) has been most consistently reported in lymphoma, while their role in leukaemia and multiple myeloma remains limited. This study investigated the humoral response to the envelope proteins of HERV-K and HERV-H in the peripheral blood of patients with multiple myeloma and non-Hodgkin lymphoma and assessed HERV-K envelope gene expression through an approach combining indirect ELISA and quantitative PCR. The study revealed an increased humoral response against the HERV-K envelope epitope in patients with non-Hodgkin lymphoma compared to matched healthy controls. However, no differences were observed in patients with multiple myeloma. Although limited to the humoral level, these findings support the relevance of HERV-K specific immune responses in non-Hodgkin lymphoma and provide a rationale for further investigation.

## Introduction

Haematological malignancies, which affect the blood, bone marrow, and lymphatic system, account for approximately 9% of all cancer cases and are commonly classified into leukaemia, lymphomas, and multiple myeloma [1, 2].

Among these, lymphoma originates from the malignant transformation of lymphocytes, potentially involving both lymphoid organs and extra-nodal sites, and is classified into Hodgkin lymphoma (HL) and non-Hodgkin lymphoma (NHL) [3, 4]. NHL is more common than HL, comprising over 40 subtypes that arise from B cells in approximately 85–90% of cases, while the remaining cases arise from T cells [5, 6]. Although the aetiopathogenesis of lymphoma is not yet fully understood, causative genetic alterations, such as chromosomal translocations, can be identified in most subtypes [2, 7]. In addition, viral agents have been implicated in lymphoma development, including Epstein–Barr virus (EBV), as EBV-infected B lymphocytes may contribute to the pathogenesis, and Human T-cell Leukaemia Virus type 1 (HTLV-1), which is associated with specific T-cell lymphomas [8, 9].

Multiple myeloma (MM) is marked by cycles of remission and relapse and is driven by the uncontrolled proliferation of monoclonal antibody-producing plasma cells [10–12]. It is usually preceded by a pre-malignant condition known as monoclonal gammopathy of undetermined significance (MGUS) [13]. Most commonly affecting the elderly population [14]. MM has been associated with several risk factor. Environmental factors, such as exposure to pesticides or herbicides, appear to increase the risk of disease development, [15, 16] while, genetic abnormalities have been implicated in its prognosis [17]. In addition, epigenetic alterations, particularly changes in DNA methylation, may contribute to increased susceptibility to MM [18].

Several studies have shown that human endogenous retroviruses (HERVs) are involved in a range of diseases, including cancer, as well as neurodegenerative and autoimmune disorders [19–23].

HERVs originate from ancient infections by exogenous retroviruses that integrated into the human germline, enabling their vertical transmission across generations [24, 25]. They account for approximately 8% of the human genome and have been implicated in both physiological and pathological processes [26, 27]. HERV-K is known to influence cancer cell proliferation by modulating immune responses and affecting cancer stem cell growth [28, 29]. Similarly, HERV-H expression has been detected in various tumours, including liver, lung, and testicular cancers, as well as in human leukaemia cell lines [30, 31].

Increased expression of HERV genes has been observed during the progression of haematological malignancies, including lymphoma, MM and leukaemia [32–34]. In particular, elevated levels of circulating viral RNA and increased HERV-K gene expression have been detected in the bloodstream of patients with NHL [33]. In MM, HERV-K expression has been found to be higher in bone marrow plasma cells of patients compared to those with MGUS and healthy controls (HCs) [34]. In line with these findings, upregulation of HERV-H gene expression has been observed in lymphoma cell lines [35].

This study aimed to evaluate the humoral response against immunogenic epitopes of the HERV-K and HERV-H envelope proteins in the plasma of patients with NHL and MM. In the NHL cohort, we also assessed the humoral response against non-HERV epitopes, specifically targeting Interferon Regulatory Factor 5 (IRF5) to investigate on general level of autoantibodies. In addition, HERV-K envelope gene expression was evaluated in peripheral blood mononuclear cells (PBMCs) from patients.

## Materials and Methods

### Patients and Samples

A total of 106 patients, diagnosed with NHL and MM were recruited from Haematology Department of University Hospital (AOU). The HCs group of 106 age and sex-matched individuals was recruited from the Transfusional Centre of Sassari, Italy.

Patients with MM and NHL were divided into two subgroups based on their clinical status at the time of the samples collection. Those who had already received therapy were classified as NHL-T and MM-T, while newly diagnosed, treatment-naive patients were classified as NHL-UT and MM-UT. Clinical-pathological and demographic data of all the participants are summarised in Table 1.

**Table 1:**
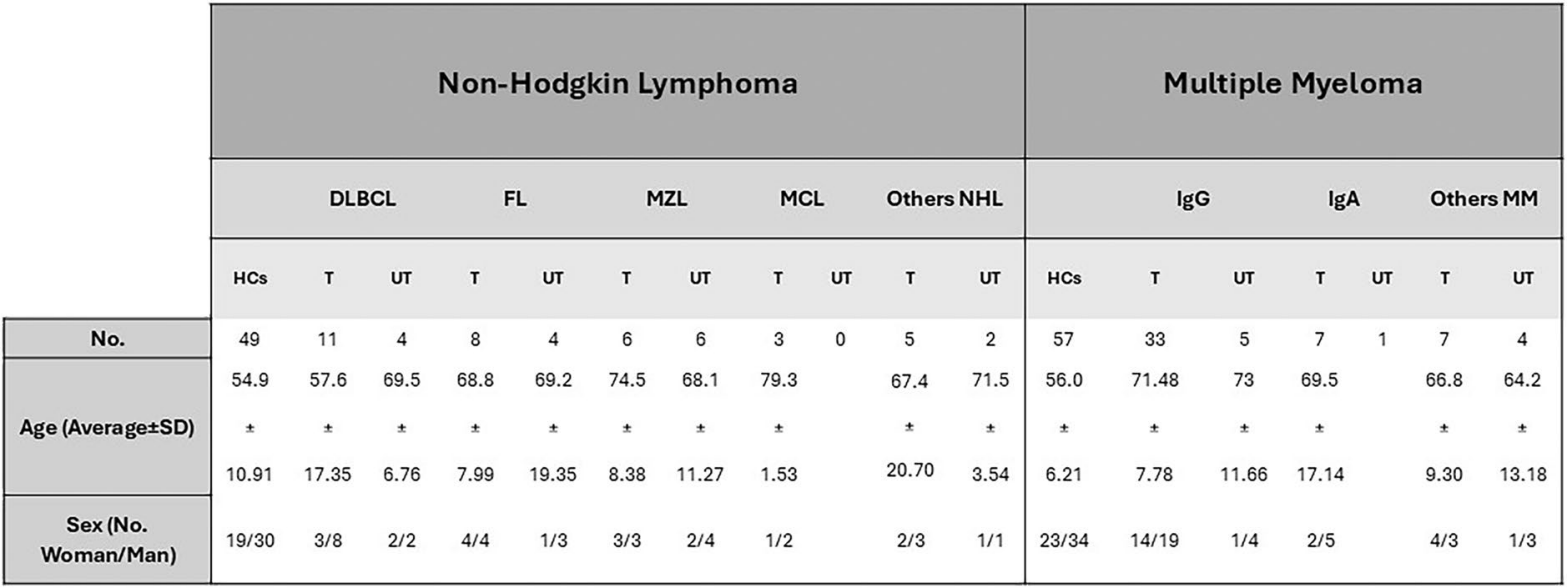
Clinical and demographic characteristics of Non-Hodgkin Lymphoma, Multiple Myeloma patients and Healthy Controls. In the table it can find the size of the population under study, the age, and the sex. The NHL patients were categorised according to diagnosis (DLBCL=Diffuse large B-cell lymphoma; FL= Follicular lymphoma; MZL= Marginal zone lymphoma; MCL= Mantel cell lymphoma and other NHL). The MM population was divided into three diagnostic subgroups: IgG, IgA, and others MM.

Plasma and PBMCs isolations from peripheral blood was performed by standard Ficoll Histopaque (Sigma-Aldrich, St. Louis, MO, USA) gradient centrifugation. The isolated plasma samples were stored at—20 ℃, while the PBMCs, isolated by density gradient centrifugation were washed twice in phosphate-buffered saline (PBS) and stored at -80 ℃ in in fetal bovine serum (FBS) supplemented with 10% dimethyl sulfoxide (DMSO).

All peripheral blood samples were processed in compliance with biosafety regulations and using appropriate personal protective equipment (PPE).

### Peptides

Synthetic peptides were selected according to their immunogenicity (IMMUNO EPITOPE DATABASE, LA JOLLA) [36]. The portion of HERV-K _env_su19-37_ (VWVPGPTDDRCPAKPEEEG) and HERV-K _env_su109-126_ (RPKGKTCPKEIPKGSKNT) from the HERV-K envelope protein (UniProt accession O42043), the HERV-H _env_su229-241_ (LGRHLPCISLHPW) from the HERV-H envelope protein (UniProt accession Q9N2J8) and IRF5 424–434 (VVPVAARLLLE) (UniProt accession Q13568) were investigated. Peptides were synthesized with purity greater than 90% and purchased from LifeTein (South Plainfield, NJ, USA). All the peptides were reconstituted at 10 mM in DMSO and stored at -20 ℃.

### Enzyme-Linked Immunosorbent Assay (ELISA)

Plasma antibodies targeting the selected antigens were detected by indirect enzyme-linked immunosorbent assay (ELISA) for immunoglobulin G (IgG), as previously described [37]. The equimolar concentrations of the peptides were set at 10 μg/mL. To ensure the validity of the experiments, a negative control using low-reactive plasma, a positive control using highly reactive plasma, and a blank control, where the immobilized peptide was incubated with the secondary antibody alone, were included. Each sample was analysed in duplicate, and reactivity was assessed by calculating the mean optical density (OD) at 405 nm.

### RNA Extraction and Quantitative PCR (qPCR)

RNA was extracted from PBMCs by RNeasy® Plus Mini Kit (Qiagen, Hilden, Germany) according to the manufacturer’s protocol. RNA concentration and quality were evaluated by Thermo Scientific™ NanoDrop 2000 spectrophotometer (Thermo Fisher Scientific). The cDNA synthesis was performed using the High-Capacity cDNA Reverse Transcription Kit, Applied Biosystems™ (ThermoFisher Scientific) and Oligo(dt)20 Primer Invitrogen™ (ThermoFisher Scientific) according to the manufacturer’s protocol. Quantitative real-time polymerase chain reactions (qPCR) were performed using the QuantiNova® Probe PCR Kit (500) (Quiagen, Hilden, Germany) in Real-Time PCR Detection System (BIO RAD). Previously published primers, probes, for HERV-K *envelope* gene detection were applied [38]. The expression analysis results were normalized relative to the housekeeping gene glyceraldehyde-3-phosphate dehydrogenase (GAPDH). Negative controls, which contained all component except the sample, were included in each assay.

### Statistical Analysis

Statistical analysis was performed using GraphPad 8.4.3 Software (GraphPad Software, San Diego, CA, USA). Data distribution was evaluated by Shapiro–Wilk test. To assess whether there were significant overall differences among the three groups, the non-parametric Kruskal–Wallis test was performed, followed by pairwise comparisons using Dunn’s post hoc test. Receiver-operating characteristic (ROC) was used to evaluate the discriminatory power of the assays. The cut-off for positivity in each assay was set at 95% specificity, and the corresponding sensitivity was calculated accordingly. A *p* value ≤ 0.05 was considered statistically significant. qPCR data were analysed using the 2^−ΔΔCt^ method[39]. Group comparisons were conducted using aforementioned test after confirming non-normal data distribution.

## Results

### Antibody Response Against HERV-K, HERV-H Envelope and IRF5 in Plasma of NHL Patients

**Fig. 1:**
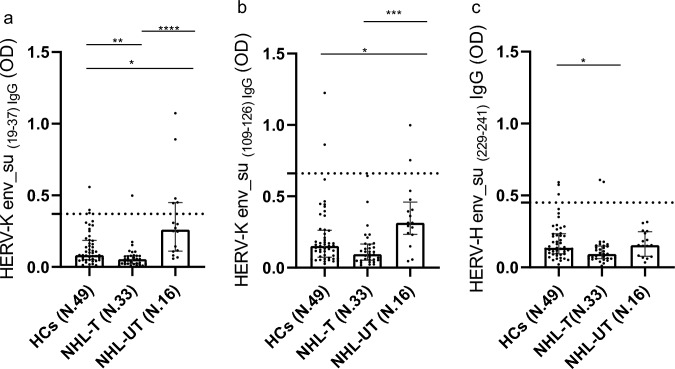
Graphs a, b and c shows the antibody response against HERV-K _env-su19-37_ (**a**), HERV-K _env-su109-126_ (**b**) and HERV-H _env 229–241_ (**c**) proteins in healthy controls (HCs), non-Hodgkin lymphoma treated (NHL-T) and non-Hodgkin lymphoma untreated (NHL-UT) patients, detect by Enzyme-Linked Immunosorbent Assay (ELISA). A statistical difference was reported when comparing UT and HCs in NHL population only for HERV-K envelope peptides under investigation. Square, bar and dashed line indicate median, interquartile range and cut-off respectively and are reported in all graphs. *p ≤ 0.05; **p < 0.01; ***p < 0.001; ****p < 0.0001

**Fig. 2:**
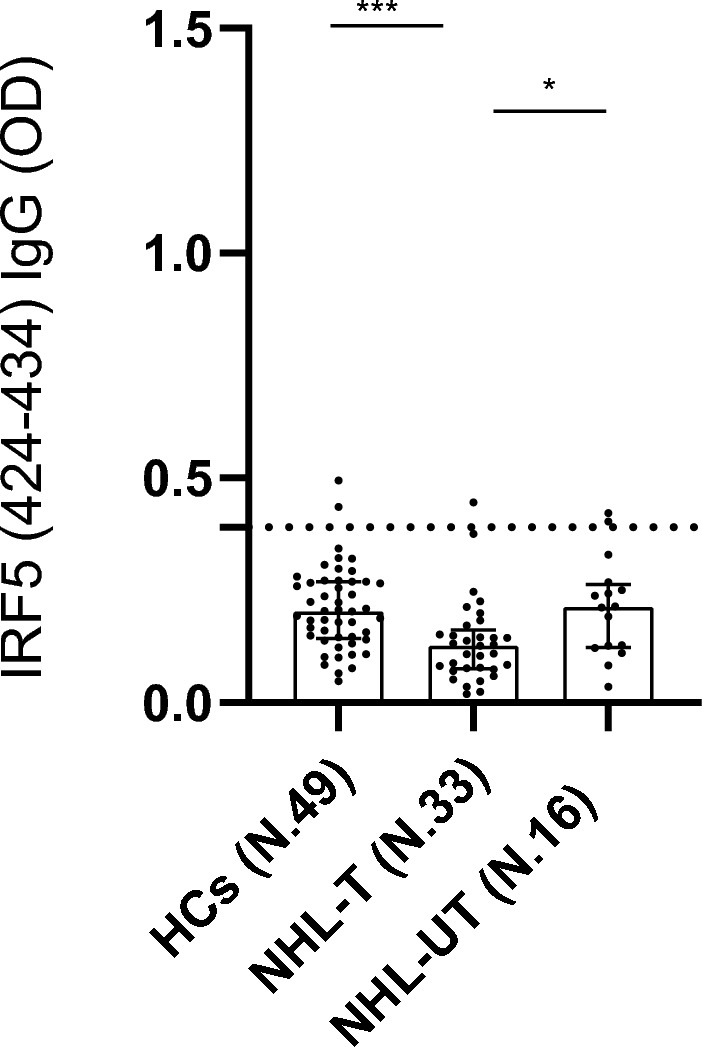
The graph shows the humoral response against IRF5 424–434 in NHL population detect by Enzyme-Linked Immunosorbent Assay (ELISA). No Statistical difference was observed between NHL-UT and HCs comparation. Square, bar and dashed line indicate median, interquartile range and cut-off respectively and are reported in the graph. *p ≤ 0.05; **p < 0.01; ***p < 0.001; ****p < 0.0001

Statistical analysis revealed significant differences in antibody levels against the HERV-K _env_su19-37_ (*p* value < 0.0001) and HERV-K _env_su109-126_ (*p* value = 0.0002), as well as the HERV-H _env_su229-241_ (*p* value = 0.0101), among NHL patients and HCs, as determined by Kruskal–Wallis test (Fig. 1). Dunn’s post hoc test indicated that NHL-UT exhibited significantly higher antibody responses to HERV-K _env_su19-37_ and HERV-K _env_su109-126_ compared to both HCs and NHL-T. In addition, HCs showed significantly higher reactivity to HERV-K _env_su19-37_ compared to NHL-T patients (Fig. 1a, b). Regarding the HERV-H _env_su229-241_, no significant difference was observed between NHL-UT and HCs, while a lower humoral response was detected in NHL-T patients compared to HCs. These results are illustrated in Fig. 1-c and detailed *p* values and statistical parameters are reported in Table 2. We also evaluated the humoral response against a non-HERV target, the IRF5 424-434 epitope. While the Kruskal–Wallis test revealed an overall significant difference among groups (*p* value = 0.0004), pairwise comparisons showed no significant differences between NHL-UT patients and HCs (Fig. 2).

**Table 2:**
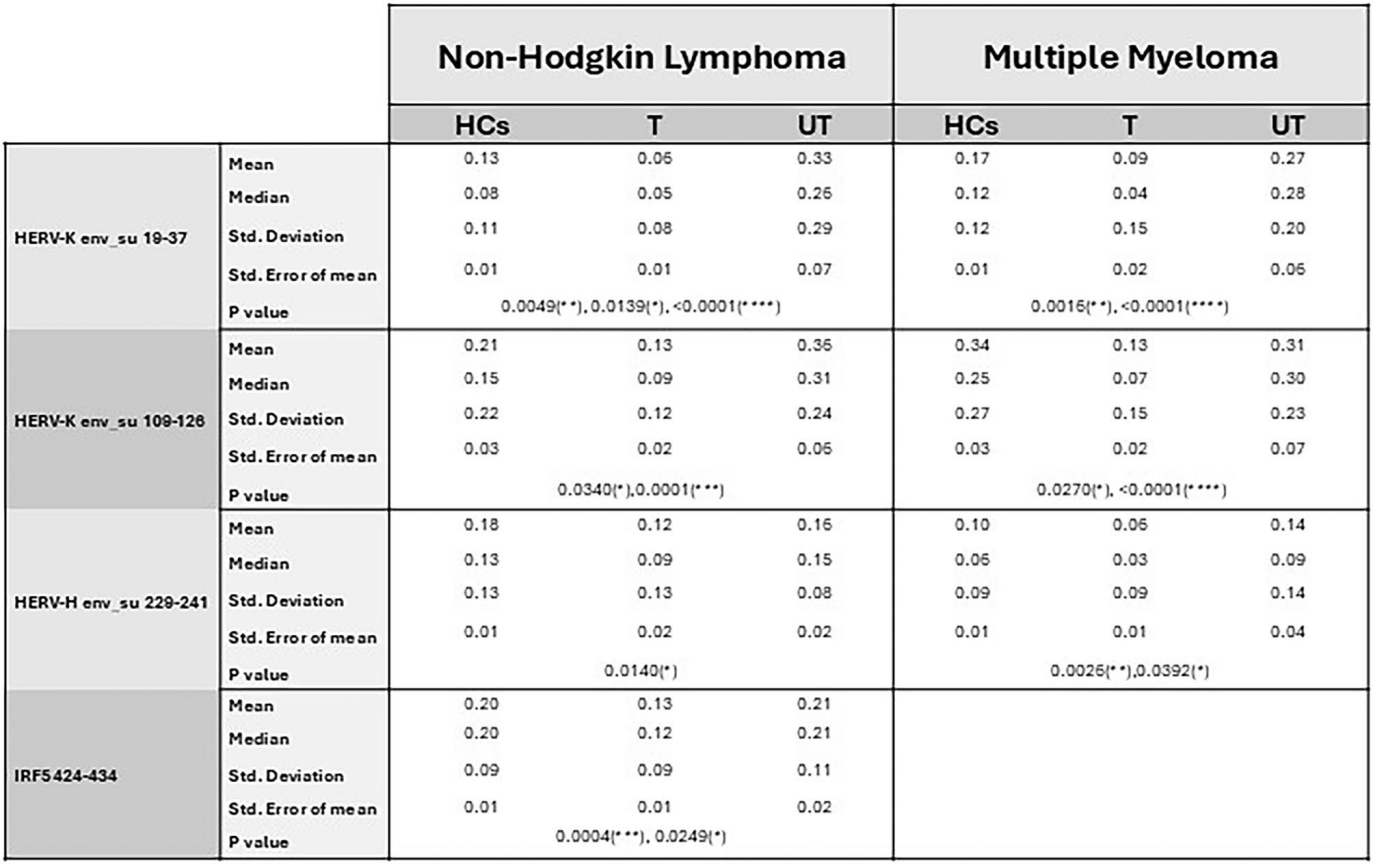
Mean statistical data for the indirect ELISA result obtained for the two population under study. The table shows the mean, median, standard deviation, standard error of mean and p value.

### Antibody Response Against HERV-K and HERV-H Envelope in Plasma of MM Patients

**Fig. 3:**
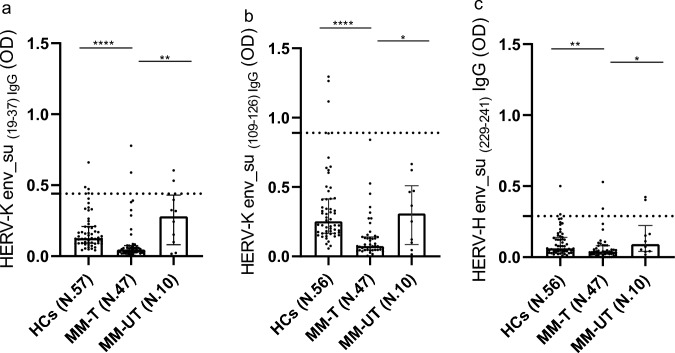
Graphs a, b and c represent humoral response against HERV-K _env-su19-37_ (**a**), HERV-K _env-su109-126_ (**b**) and HERV-H _env_su229-241_ (**c**) proteins in healthy controls (HCs,) multiple myeloma treated (MM-T) and multiple myeloma untreated (MM-UT) patients, detect by Enzyme-Linked Immunosorbent Assay (ELISA). Square, bar and dashed line indicate median, interquartile range and cut-off respectively and are reported in all graphs. *p ≤ 0.05; **p < 0.01; ***p < 0.001; ****p < 0.0001

We observed differences in antibody levels against the HERV-K _env_su19-37_ (*p* value < 0.0001) and HERV-K _env_su109-126_ (*p* value < 0.0001), as well as the HERV-H _env_su229-241_ (*p* value = 0.0012), between MM patients and HCs, as determined by Kruskal–Wallis test (Fig. 3a–c). Dunn’s post hoc test showed higher antibody response in MM-UT compared to MM-T, but no significant increase relative to HCs. Conversely, MM-T patients exhibited lower antibody titres against all the epitopes compared to HCs. Specific *p* value and additional statistical results for MM population are summarized Table 2.

### Gene Expression of HERV-K Envelope in PBMCs of NHL and MM Patients

**Fig. 4:**
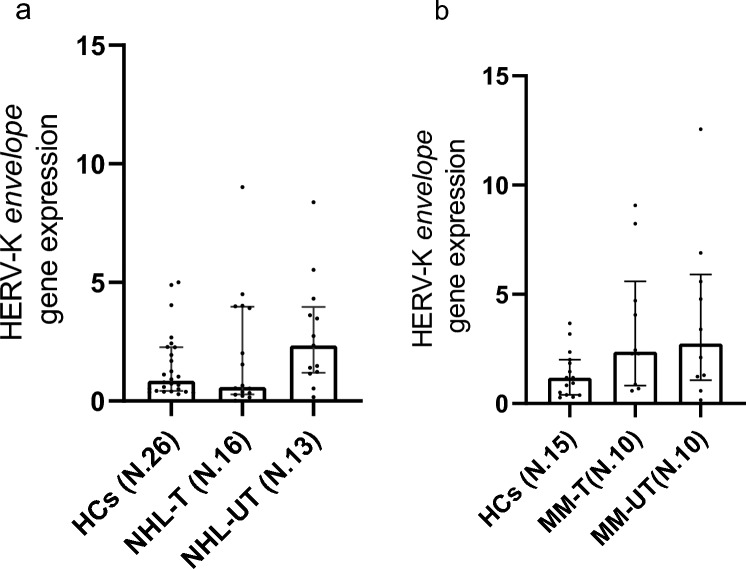
Gene expression of HERV-K *envelope* gene in the PBMCs of healthy controls (HCs), non-Hodgkin lymphoma treated (NHL-T), non-Hodgkin lymphoma untreated (NHL-UT) and HCs, (**a**), multiple myeloma treated (MM-T) and multiple myeloma untreated (MM-UT) patients (**b**) was detected by quantitative PCR (qPCR). Square, bar and indicate median, interquartile range respectively and are reported in all graphs. No differences were observed between NHL-UT, NHL-T and HCs gene expression comparison. The same trend was observed for MM population

qPCR was performed on PBMCs from NHL patients, however the Kruskal–Wallis test did not reveal any statistically significant differences (*p* value = 0.1373) (Fig. 4a). Similarly, qPCR analysis on PBMCs sample from MM patients showed no significant differences (*p* value = 0.0559) (Fig. 4b).

## Discussion

This study analysed the humoral response against specific immunogenic envelope protein epitopes of HERV-K and HERV-H in the plasma of patients with NHL and MM. Notably, NHL-UT showed elevated antibody responses to HERV-K envelope epitopes. In contrast, a significant reduction in anti-HERV-K antibody titres was observed in NHL-T and MM-T, suggesting that therapy may modulate the humoral immune response, potentially through immunosuppressive mechanisms.

These findings are consistent with previous studies that have reported both upregulated HERV-K gene expression and increased humoral reactivity in several solid tumours, including breast, prostate, ovarian cancer, and melanoma [40–42].

In lymphoma specifically, viral RNA derived from HERV-K elements has been detected in the bloodstream [31] indicating transcriptional activity and the potential release of viral-like particles or fragments into the circulation [33]. Although NHL tumour cells are known to evade immune surveillance through various mechanisms, [43] the presence of viral RNA and other HERV-K-derived elements, recognized as non-self by the host, may elicit an antibody-mediated immune response. This could help explain the elevated anti-HERV-K antibody levels observed in NHL-UT in the absence of an increase in HERV-K gene expression. A possible contributing factor to this discrepancy is the involvement of environmental cofactors in lymphoma pathogenesis, such as herpesvirus infections, which have been shown to modulate host epigenetics and immune reactivity [8, 9, 44–46]. In particular, EBV has been reported to induce hypomethylation at the long terminal repeats (LTRs) of several HERV families, thereby enhancing HERV transcription and facilitating B-cell transformation [47]. Additionally, it has been shown to transactivate HERV-K18, whose product may act as a superantigen, [48] leading to a non-specific activation of B cells and enhanced antibody production [49]. Correlations between EBV infection and HERV activation have also been observed in other immune-mediated diseases, such as multiple sclerosis [50]. In this context, the robust humoral response observed against HERV-K in NHL-UT patients, despite the lack of elevated HERV-K gene expression, may be partially explained by a mechanism involving superantigen-like activity.

These findings suggest that the humoral response to HERV-K envelope proteins may serve as a biomarker for NHL, with potential relevance for early disease detection, as already proposed in some solid tumours [42]. The lack of increased antibody reactivity to a non-HERV antigen (IRF5) in NHL-UT patients compared to HCs supports the specificity of this response and argues against a generalised immune activation [41]. Moreover, the reduction in humoral responses in NHL-T patients likely reflects the immunomodulatory effects of therapeutic regimens, such as chemo-immunotherapy. [51] Although previous studies have implicated a synergistic role for HERV-K and HERV-Fc1 loci in MM pathogenesis, [52] and Masuda et al. reported elevated HERV-K RNA expression in plasma cells and MM-derived cell lines, [34] our data did not show a significant increase in humoral reactivity in MM-UT compared to HCs. This finding may reflect the inherently immunosuppressive microenvironment of MM, which can be present even in the early stages of disease [53]. The further decrease in antibody levels observed in MM-T likely results from the combined effects of disease progression and treatment-related immune suppression [53, 54]. A similar trend was observed in the gene expression analysis, further confirming the lack of difference seen in the humoral response.

## Conclusion

These results support the idea that autoantibodies against the HERV-K envelope could be explored as potential biomarkers for NHL, with possible relevance in the diagnostic setting. In MM, however, different approaches may be needed to investigate the role of HERV-K. Given the immunosuppressive features of the disease, assessing humoral responses may not be the most appropriate or informative strategy for biomarker development.
